# Rapid assessment of heavy metal pollution using ion-exchange resin sachets and micro-XRF core-scanning

**DOI:** 10.1038/s41598-019-43015-x

**Published:** 2019-04-29

**Authors:** Jyh-Jaan Steven Huang, Sheng-Chi Lin, Ludvig Löwemark, Sofia Ya Hsuan Liou, Queenie Chang, Tsun-Kuo Chang, Kuo-Yen Wei, Ian W. Croudace

**Affiliations:** 1 Department of Geosciences, National Taiwan University, Taipei, Taiwan; 2 Research Center of Future Earth, National Taiwan University, Taipei, Taiwan; 3 Institute of Geology, University of Innsbruck, Innsbruck, Austria; 4 Center for Teaching Excellence, National Pingtung University of Science and Technology, Pingtung, Taiwan; 5 Center of Integrative Geosciences, University of Connecticut, Storrs, Connecticut USA; 6 Department of Bioenvironmental Systems Engineering, National Taiwan University, Taipei, Taiwan; 7 GAU-Radioanalytical, University of Southampton, National Oceanography Centre, Southampton, United Kingdom

**Keywords:** Environmental monitoring, Sustainability

## Abstract

Conventional pollution monitoring strategies for heavy metals are often costly and unpractical. Innovative sampling and analytical approaches are therefore needed to efficiently monitor large areas. This study presents a novel, simple, fast, and inexpensive method to monitor heavy metal pollution that uses cation-exchange resin sachets and the micro-XRF core-scanning technique (XRF-CS). The resin passive samplers act as concentrators of cationic species and can be readily deployed spatially and temporally to record pollution signals. The large number of analytical tasks are then overcome by the fast and non-destructive XRF-CS to precisely assess elemental concentrations. Quantifying element loading involves direct comparison with a set of identically prepared and scanned resin reference standards containing Ca, Ti, Cr, Mn, Ni, Cu, Zn, Pb. The results show that within the test range (from 0–1000 s mg kg^−1^), the calibration lines have excellent regressions (R^2^ ≥ 0.97), even at the shortest exposure time (1 s). A pilot field survey of a suspected polluted area in central Taiwan, where 30 resin sachets had been deployed, identified a pollution hot spot in a rapid and economical manner. Therefore, this approach has the potential to become a valuable tool in environmental monitoring and forensics.

## Introduction

Environmental pollution arising from industrial activities is a worldwide concern that threatens public health and socio-economic sustainability^1–3^. One of the most important issues is the pollution caused by industrial use of heavy metals. When released into the environment, these toxic and persistent metals are hazardous to aquatic ecosystems and will ultimately threaten public health due to accumulation in the food chain^4,5^. However, the conventional strategy for water pollution monitoring is based on the collection of discrete water samples at prescribed times with subsequent instrumental analyses^4^. Such strategies are costly and often unpractical since illicit wastewater discharges are clandestine and irregular^6^. Pollutant concentrations can also have variable spatial distributions due to sporadic discharges into complex local drainage systems making sampling difficult^7,8^. Developing a time and cost-efficient way of monitoring heavy metals over large areas is therefore challenging and requires innovative sampling and analytical approaches.

Passive and integrating sampling methods that can capture anomalous metal concentrations are suitable for initial monitoring in water bodies with fluctuating pollutant levels^9–12^. Such methods also have the potential to identify discharge points for further and more detailed investigation. As one of the receiving-phase materials, ion-exchange resin has been developed as an economically feasible method for extracting heavy metals in wastewaters over the past decades, even at low concentration effluents^13–15^. The rapid sorption characteristics of ion-exchange resins^16^ and their low cost make resin samplers an effective way to provide an integrated record of pollution over the sampler deploying time. Measurement of the metal concentrations in the resin samplers does commonly involve elution of the absorbed metals followed by analysis such as AAS or ICP-MS. However, chemical extraction and analysis procedures of resin can be slow, costly, and time-consuming. We therefore introduce the fast, non-destructive, and multi-element micro X-ray fluorescence core-scanning technique (XRF-CS) to overcome these restrictions.

Micro XRF-core scanners have revolutionized the analysis of sediment cores over the last decade^17^. This fast and high-resolution technique is used to rapidly assess elemental variations for a wide array of research topics, including environmental forensics and anthropogenic influences^18–20^. However, the XRF-CS results, usually represented as elemental counts, are often considered as semi-quantitative measurements due to possible X-ray absorption or scattering caused by down core variabilities of the scanned materials^21,22^. Although many studies have attempted to calibrate scanning counts into concentrations by different methods^22,23^, case-by-case calibrations are generally needed due to the heterogeneity in each sampling location, resulting in variations in the sample matrix. The environmental applications of XRF-CS thus are limited, especially for pollution studies which generally require quantitative concentrations.

By using ion-exchange resin as a pollutant trap in combination with XRF-CS, the dry and homogenous resin samples, having the same composition, will minimize the matrix effects which normally impact on XRF-CS analyses. Therefore, this study has developed a cost-effective, readily-deployable ion-exchange resin sachet as the integrating passive sampler. Multiple samplers can be easily installed and left for suitable times depending on the motivation of the monitoring program. The sensitive and non-destructive XRF-CS can therefore fulfill the demand to quantitatively assess heavy metal concentrations of the resin samples in a fast and economical manner, which allows for archiving samples of potential legal significance (Fig. S1).

To test the potential of this approach, resin reference standards were prepared under ideal laboratory conditions to systematically investigate the performance of XRF-CS. The regression lines and correlation coefficients of elements that are generally of interest in pollution studies (Cr, Mn, Ni, Cu, Zn, Pb), as well as elements common in natural waters (Ca, Ti), were examined and tested using different XRF exposure times. In addition, to test the feasibility of this approach in a real aqueous environment with complex constituents, a pilot field monitoring was performed in a suspected polluted area in central Taiwan. The result, combined with a geographic information system (GIS) tool, helped to identify the hot zone of heavy metal pollution over a large area in a fast and economical manner, contributing to the developments of environmental monitoring and forensics.

## Materials and Methods

### Preparation of resin reference standards

The cation-exchange resin Amberlite^TM^ IR-120 (Rohm and Hass, USA) was used as the solid sorbent in this study. The physical and adsorptive properties of the resin have been reported by previous works^14,16^. 20 g of the resin, in Na^+^ form, was filled into a polyethylene (PE) net bag and sealed to produce the resin sachet (Fig. S2a). Four standard solutions were prepared by diluting commercial stock solutions (1000 mg/L, Sigma-Aldrich^®^) of Cr, Mn, Ni, Cu, Zn, Pb, Ca, Ti (Ca and Ti were added to simulate the common elements in natural water) with deionized water according to the EPA effluent standards of Taiwan (Table S1). To further simulate different flow rates in the natural environment, the four standard solutions were combined with four stirring speeds (50, 100, 150, 200 rpm, performed by Jar-Tester) and separated into 16 batch sets of 1 L volume, respectively. The resin sachets were put in each set for 2 hours at 293 K to produce the resin reference standards for further Itrax XRF-CS measurements. 5 ml of each remaining solutions were then analyzed by ICP-OES (Thermo Scientific iCAP7000) for metal concentrations. The concentrations of the resin reference standards were then determined by mass balance from initial and final concentrations of the standard solutions, according to the following equation:

1 $${{\rm{C}}}_{m}=\frac{({C}_{0}-{C}_{t})\ast L}{w}\ast 1000$$

Where *C*_*m*_ represents the concentration of the resin reference standards (mg kg^−1^); *C*_0_ and *C*_*t*_ are the initial and final concentrations of metal in the standard solutions (mg *L*^−1^), respectively. L and w are the volume (L) of the standard solutions and the weight of resin (g).

### Itrax XRF-CS of resin reference standards

The 16 lab-prepared resin reference samples plus one blank resin sample (in total 17 resin reference standards) were rinsed with deionized water, dried at room temperature, placed in 2 × 2 × 2 cm custom-made plastic sample cups (similar to the sample cups described in a previous study^24^), and finally covered by a 1.4 μm PE X-ray Film (Cox Analytical Systems, Gothenburg) to avoid potential contamination and static interference. Seventeen resin reference standards were analyzed using the Itrax micro-XRF core scanner (Mo X-ray tube with 30 kV, 22 mA, 1 mm scanning resolution, and 100 s exposure time) at the Department of Geosciences, National Taiwan University (NTU) and compared with their absolute concentrations (obtained by Equation 1). In addition, five resin reference standards from low to high concentrations have been further scanned at different exposure times (30 kV, 31 mA, 1 mm scanning resolution with 1, 5, 15, 30, 100 s exposure time, respectively) to address the influence of exposure time on the accuracy of the measurements. All resulting XRF spectra were evaluated by the Q-Spec software (version 8.6.0., COX Analytical System, Gothenburg) to convert the elemental peak areas into counts. Each resin reference standard was measured at 20 points as the scan tracked across the 2 cm wide sample cup at 1 mm scanning resolution. Only 10 of these 20 continuous measurements in the central part were selected to exclude edge effects caused by the sample cups. The averages and standard deviations of the XRF-CS counts were subsequently calculated and compared with measurements made on the standards (Equation 1). Therefore, the linear regression lines and correlation coefficients (R^2^) of Cr, Mn, Ni, Cu, Zn, Pb, Ca and Ti can be determined, respectively.

### Pilot field survey

In Taiwan, as a result of rapid industrial expansion in the past seven decades, illegal discharges of industrial wastewaters have caused widespread and severe heavy metal pollution to aquatic and terrestrial systems^25,26^. Today, about 20% of the arable land in Taiwan (1.7*10^5^ ha) is considered to be affected by heavy metal pollution. Approximate 400 ha of farmland is categorized as restricted by the Taiwan Environmental Protection Administration (EPA) according to the current soil quality standards^27^. In this study, a pilot field survey was conducted in the north of Changhua County, central Taiwan. Due to poor land-use planning, many factories, including electroplating, metalwork, and metal surface treatment industries, are located in the agricultural area and have been suspected of discharging wastewater into irrigation channels^7,25^. With the high density of irrigation channels (~1.06 km/ha)^25^ and fluctuating pollutant concentrations, it is difficult to trace the pollution source and behavior by conventional monitoring strategies. The area therefore provides a suitable field site for testing our novel approach.

The field monitoring device consists of a resin sachet protected by a commercial plastic sink filter with a 10-meter long fishing line (Fig. S2b). At the cost of less than 1 US$ each, 30 devices were deployed into the selected irrigation channels for 7 days in a 12 km^2^ monitoring area. When retrieved, the resin samples were washed, dried, and analyzed by the Itrax XRF-CS applying the aforementioned procedure with a Mo-tube in the setting of 30 kV, 22 mA, 1 mm scanning resolution and 100 s exposure time. The resulting XRF-CS counts of Cr, Mn, Ni, Cu, Zn, and Pb of each monitoring location were plotted by Arc GIS 10.2 to show the spatial distribution of each pollutant, respectively.

## Results and Discussion

### XRF-CS results of resin reference standards

**Figure 1:**
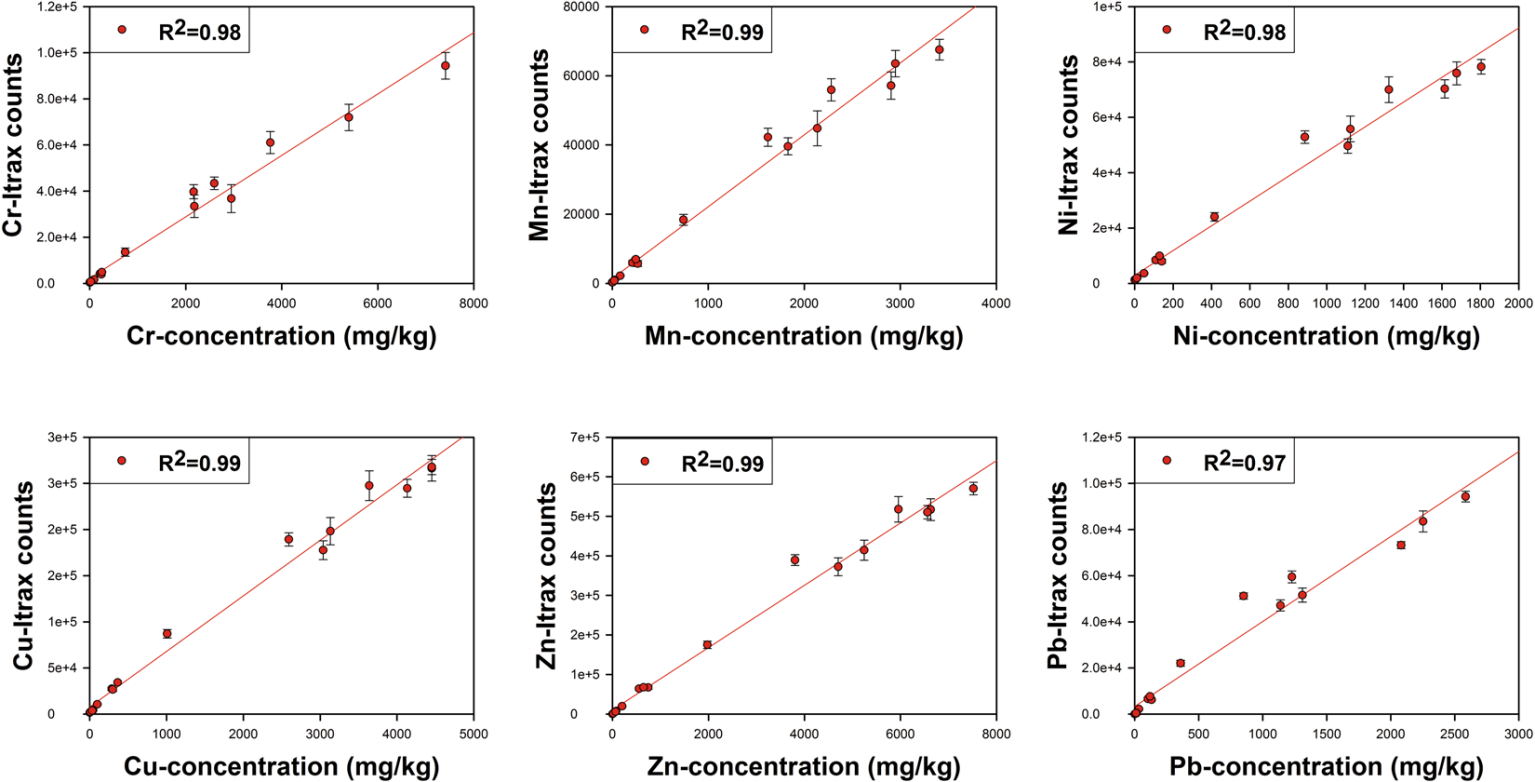
Scatter plots showing linear regression lines and correlation coefficients between concentrations and XRF-CS counts (100 s exposure time, with the standard deviation of each measurement) of resin reference standards for elements generally used in pollution studies (Cr, Mn, Ni, Cu, Zn, Pb).

Due to different combinations of standard solutions and stirring speeds, the 17 resin reference standards cover the concentration range from few ppm to thousands of ppm for each tested metal (Fig. 1, X-axis). All the tested elements, including the pollution-related Cr, Mn, Ni, Cu, Zn, Pb and the nature-related Ca, Ti, show excellent linear correlations (R^2^ ≥ 0.97) between XRF-CS counts and absolute concentrations calculated by Equation 1 (Fig. 1 for Cr, Mn, Ni, Cu, Zn, Pb and Fig. S3 for Ca, Ti). Such a finding also demonstrates that, compared to the sediment which is the originally designed matrix for the micro-XRF core scanner, dry and homogenous resins run well and enable easy application of the XRF-CS technique.

### Exposure time experiment

**Figure 2:**
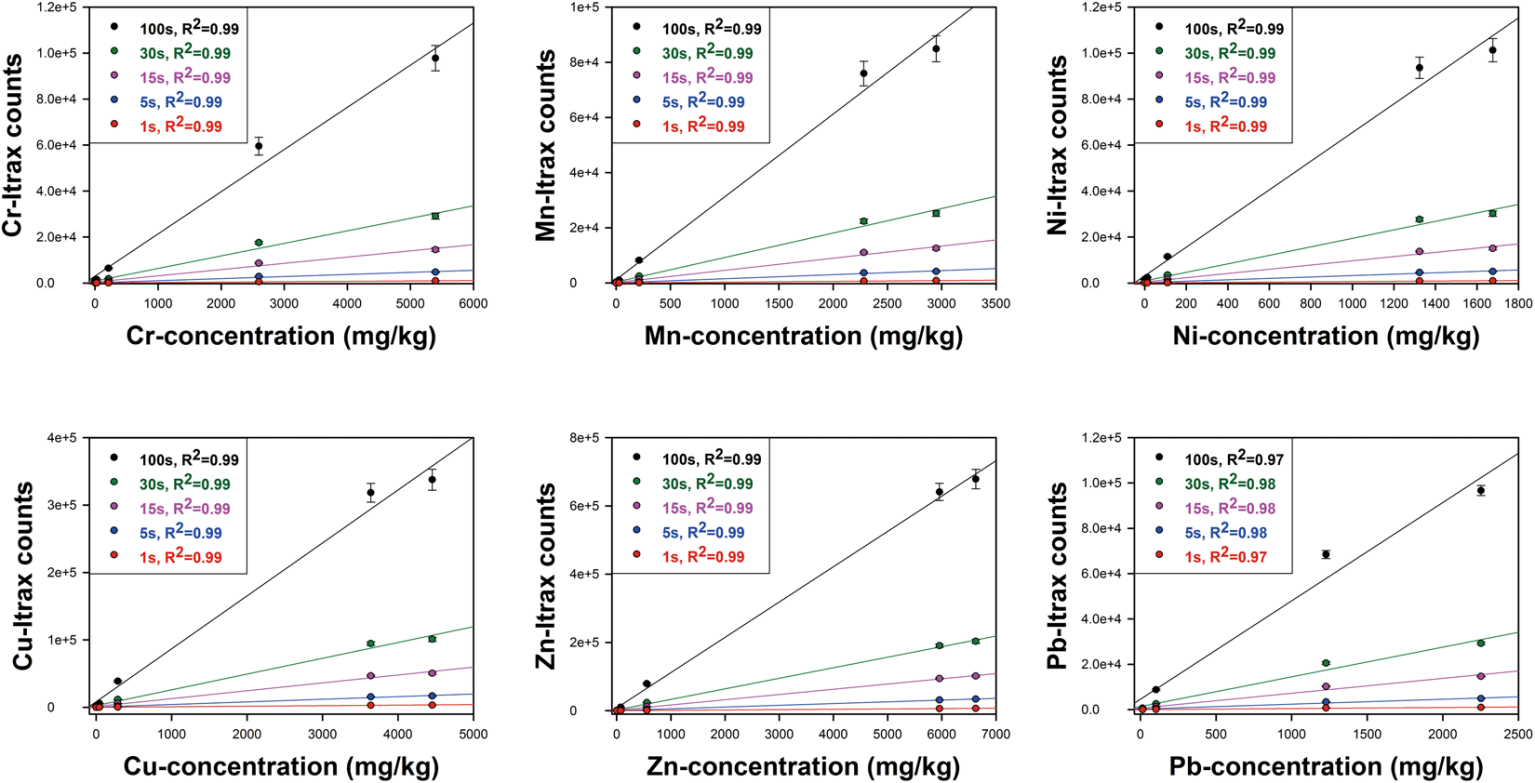
Combined results of concentrations vs. XRF-CS counts for five tested exposure times. Correlation coefficients of all experiments are ≥0.97, suggesting the chosen exposure times have only a limited influence on the accuracy of XRF-CS results.

Exposure times of 100 s per measuring point is a common setting for the Itrax XRF-CS when analyzing discrete samples^28^. With this setting, each resin sample needs around 30 minutes to finish the measurement (i.e. 20 points measured across a 20 mm of sample cup at 1 mm scanning resolution). Although this is already rather short and convenient compared to conventional chemical extraction and analysis procedures, the measurement time may be unnecessarily long for the environmental monitoring purpose. It was shown by Huang *et al*.^29^ that long exposure times have only minor effects on the accuracy of XRF-CS measurements. Our exposure time experiments, with five resin reference standards from low to high concentrations and five exposure times from 1 s to 100 s, also show excellent correlation coefficients (R^2^ ≥ 0.97) between scanning counts and absolute concentrations (Fig. 2 for Cr, Mn, Ni, Cu, Zn, Pb and Fig. S4 for Ca, Ti). Therefore, since all the pollution-related elements have high detectability with the XRF-CS technique and are expected to have high concentrations in the wastewater, 100 s exposure time is not necessary and can be further shortened, as suggested by the previous study^29^. Moreover, since standard deviations of all tested elements with different exposure times are rather small (Figs 1, 2, S3 and S4), the homogenous resin samples can be scanned in a lower scanning resolution with automatic spectrum picking software to further lower the measurement time, similar to the procedure proposed by Ohlendorf^30^. Thus, each resin sample can be measured in approximately 20 seconds (1 s per point across a 20 mm sample cup), which is considerably shorter than the general settings of portable XRF devices (>100 s)^31^.

### Pilot field survey

**Figure 3:**
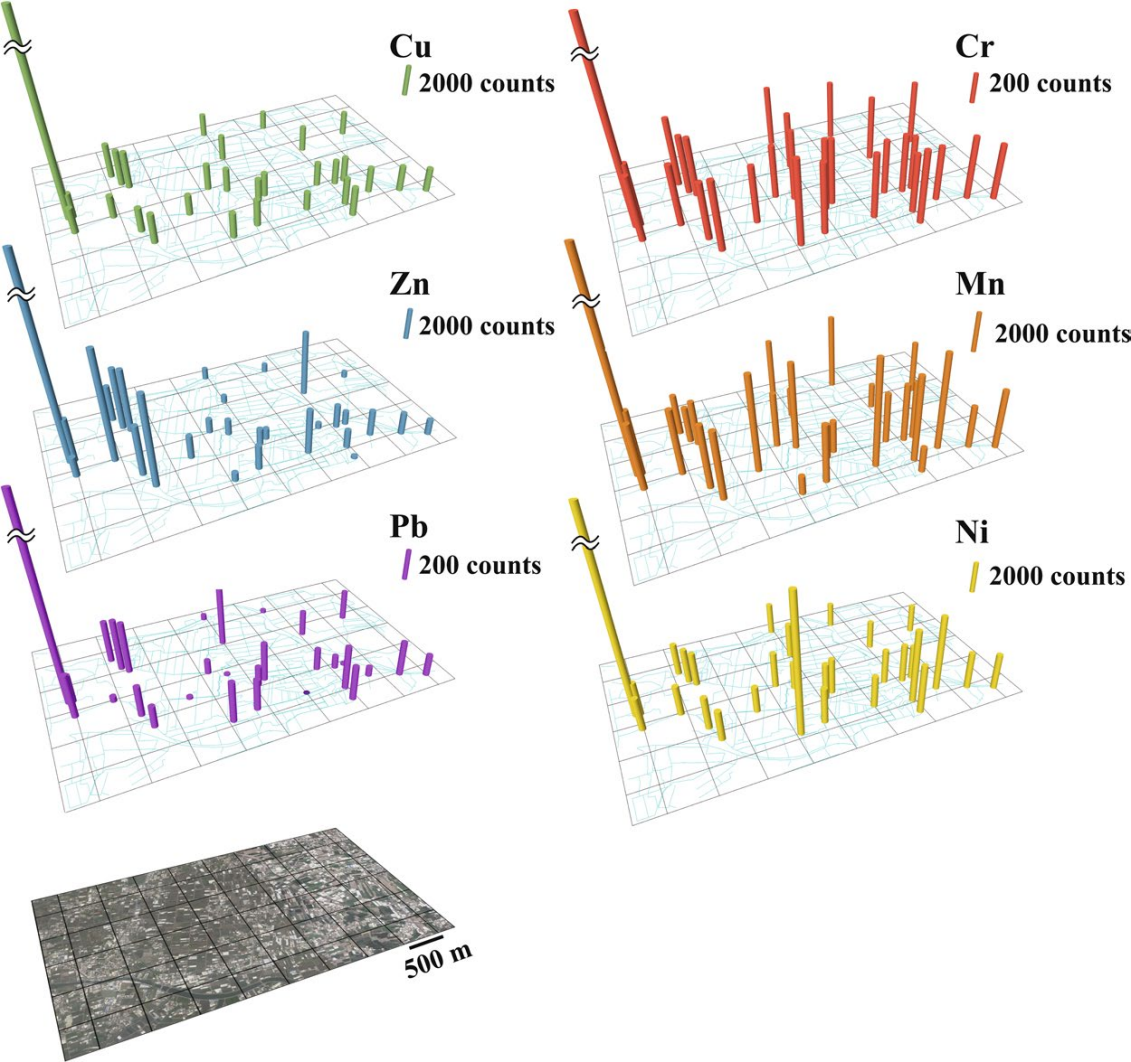
A pilot field survey with 30 resin samples in a suspected polluted farm area in central Taiwan. The height of the bars represents variations for pollution-related elements (Cr, Mn, Ni, Cu, Zn, Pb) in XRF-CS counts across the monitoring area. Each black cell represents an area of 0.5 × 0.5 km; the pollution hot spot is identified in the upper left of the grid. The satellite image was taken on 19^th^ November 2012 from DigitalGlobe with a Google Earth platform and further modified by Arc GIS 10.2.

To demonstrate the advantages and feasibility of XRF-CS approach with the resin sachets, 30 field monitoring devices were deployed for 7 days, recovered, and measured. The XRF-CS counts of pollution-related Cr, Mn, Ni, Cu, Zn, and Pb have been plotted over the local drainage area. A pollution hot spot, with very high counts for all pollution-related elements, was identified in the northwestern corner of the monitored area (Fig. 3). This finding may be linked to the illegal wastewater discharge by local metal and electroplating industries since this area is known as “the hometown of the faucet” and famous for producing hardware products. Such suspected pollution activity must have occurred during the deployment period. There was no report from conventional monitoring stations operated by the local environmental authority, probably due to the complex local drainage system and fluctuating pollutant concentrations. Therefore, by using ion-exchange resin as the passive sampling sorbent in combination with XRF-CS technique, a fast (minutes to seconds), low cost, multi-element (Si to U can be detected by the Itrax XRF-CS technique depending on the choice of the resin), and practical monitoring approach for heavy metal pollution can be used as an environmental monitoring tool for large geographic areas.

### Future outlooks of the resin sachets and XRF-CS approach

The described sampling and analytical method is an effective tool that can be adopted by regulatory organizations (local governments and environmental authorities) to record and study pollution given its effectiveness, rapidity, and relative simplicity. It is also useful for long-term wastewater management and sustainable environment governance. By adding a tamper-proof and automatic sampler with timer and GPS locator, this system could become attractive to regulatory groups and would extend the range of use. Furthermore, by combining this method with multivariate statistics (e.g., Principal component analysis, PCA), the approach could also be used in regional geochemical studies and environmental forensics to trace the source, transportation, and deposition of pollutants based on their unique chemical fingerprints.

## Supplementary information

Supplementary Information

## Data Availability

All data included in this study are available upon request by contact with the corresponding authors.

## References

[1] Croudace, I. W., Romano, E., Ausili, A., Bergamin, L. & Rothwell, R. G. X-Ray Core Scanners as an Environmental Forensics Tool: A Case Study of Polluted Harbour Sediment (Augusta Bay, Sicily) in *Micro-XRF**Studies of Sediment Cores* (eds Croudace, I. W. & Rothwell, R. G.) 393–421 (Springer, 2015).

[2] Diamond ML, Hodge E (2007). Urban contaminant dynamics: from source to effect. Environ. Sci. Technol..

[3] Wang X, Zhang F (2018). Effects of land use/cover on surface water pollution based on remote sensing and 3D-EEM fluorescence data in the Jinghe Oasis. Sci Rep.

[4] Aragay G, Pons J, Merkoci A (2011). Recent trends in macro-, micro-, and nanomaterial-based tools and strategies for heavy-metal detection. Chem. Rev..

[5] Lu, M. *et al*. Graphene Aerogel-Metal-Organic Framework-Based Electrochemical Method for Simultaneous Detection of Multiple Heavy-Metal Ions. *Anal. Chem., in press*, 10.1021/acs.analchem.8b03764 (2018). 10.1021/acs.analchem.8b03764 30338985

[6] Rozemeijer J (2010). Application and evaluation of a new passive sampler for measuring average solute concentrations in a catchment scale water quality monitoring study. Environ. Sci. Technol..

[7] Lin Y-P, Chang T-K, Shih C-W, Tseng C-H (2002). Factorial and indicator kriging methods using a geographic information system to delineate spatial variation and pollution sources of soil heavy metals. Environ. Geol..

[8] Yang X, Liu Q, Luo X, Zheng Z (2017). Spatial Regression and Prediction of Water Quality in a Watershed with Complex Pollution Sources. Sci Rep.

[9] Allan IJ (2007). Evaluation of the Chemcatcher and DGT passive samplers for monitoring metals with highly fluctuating water concentrations. J. Environ. Monit..

[10] Vrana B (2005). Passive sampling techniques for monitoring pollutants in water. TrAC, Trends Anal. Chem..

[11] Shaw M, Mueller JF (2009). Time integrative passive sampling: how well do chemcatchers integrate fluctuating pollutant concentrations?. Environ. Sci. Technol..

[12] Charriau A (2016). Overview of the Chemcatcher(R) for the passive sampling of various pollutants in aquatic environments Part A: Principles, calibration, preparation and analysis of the sampler. Talanta.

[13] Demirbas A, Pehlivan E, Gode F, Altun T, Arslan G (2005). Adsorption of Cu(II), Zn(II), Ni(II), Pb(II), and Cd(II) from aqueous solution on Amberlite IR-120 synthetic resin. J. Colloid Interface Sci..

[14] Franco PE (2013). Nickel(II) and zinc(II) removal using Amberlite IR-120 resin: Ion exchange equilibrium and kinetics. Chem. Eng. J..

[15] Carmona M, Warchoł J, Lucas A (2008). d. & Rodriguez, J. F. Ion-exchange equilibria of Pb^2+^, Ni^2+^, and Cr^3+^ ions for H^+^ on Amberlite IR-120 resin. J. Chem. Eng. Data.

[16] Jha MK, Van Nguyen N, Lee JC, Jeong J, Yoo JM (2009). Adsorption of copper from the sulphate solution of low copper contents using the cationic resin Amberlite IR 120. J. Hazard. Mater..

[17] Rothwell, R. G. & Croudace, I. W. Twenty years of XRF core scanning marine sediments: What do geochemical proxies tell us in *Micro-XRF**Studies of Sediment Cores* (eds Croudace, I. W. & Rothwell, R. G.) 25–102 (Springer, 2015).

[18] Huang, J.-J. *et al*. Disentangling natural and anthropogenic signals in lacustrine records: An example from the Ilan Plain, NE Taiwan. *Front. Earth Sci*. **4**, 10.3389/feart.2016.00098 (2016).

[19] Croudace, I. W., Teasdale, P. A. & Cundy, A. B. 200-year industrial archaeological record preserved in an Isle of Man saltmarsh sediment sequence: Geochemical and radiochronological evidence. *Quat. Int*., 10.1016/j.quaint.2018.09.045 (2018).

[20] Miller H (2014). A 500 year sediment lake record of anthropogenic and natural inputs to Windermere (English Lake District) using double-spike lead isotopes, radiochronology, and sediment microanalysis. Environ. Sci. Technol..

[21] Tjallingii R, Rohl U, Kolling M, Bickert T (2007). Influence of the water content on X-ray fluorescence core-scanning measurements in soft marine sediments. Geochem. Geophys. Geosyst..

[22] Longman, J., Veres, D. & Wennrich, V. Utilisation of XRF core scanning on peat and other highly organic sediments. *Quat. Int*., 10.1016/j.quaint.2018.10.015 (2018).

[23] Weltje, G. J. *et al*. Prediction of geochemical composition from XRF-core-scanner data: A new multivariate approach including automatic selection of calibration samples and quantification of uncertainties in *Micro-XRF**Studies of Sediment Cores* (eds Croudace, I. W. & Rothwell, R. G.) 507–534 (Springer, 2015).

[24] Itrax-Operators *et al*. Practical guidelines and recent advances in the Itrax XRF core-scanning procedure. *Quat. Int*., 10.1016/j.quaint.2018.10.044 (2018).

[25] Cheng BY, Fang WT, Shyu GS, Chang TK (2012). Distribution of heavy metals in the sediments of agricultural fields adjacent to urban areas in Central Taiwan. Paddy Water Environ.

[26] Römkens PF (2009). Characterization of soil heavy metal pools in paddy fields in Taiwan: chemical extraction and solid-solution partitioning. J. Soils Sediments.

[27] EPA. *Database of Soil and Groundwater Pollution Remediation Funds*, https://sgw.epa.gov.tw/Public/ (2018).

[28] Profe J, Wacha L, Frechen M, Ohlendorf C, Zolitschka B (2018). XRF scanning of discrete samples – A chemostratigraphic approach exemplified for loess-paleosol sequences from the Island of Susak, Croatia. Quat. Int..

[29] Huang J-J (2016). Choosing optimal exposure times for XRF core-scanning: Suggestions based on the analysis of geological reference materials. Geochem. Geophys. Geosyst..

[30] Ohlendorf C (2017). A sample carrier for measuring discrete powdered samples with an ITRAX XRF core scanner. X-Ray Spectrom..

[31] Hagiwara K, Koike Y, Aizawa M, Nakamura T (2015). On-site quantitation of arsenic in drinking water by disk solid-phase extraction/mobile X-ray fluorescence spectrometry. Talanta.

