# Supplementary material for: Rapid assessment of heavy metal pollution using ion-exchange resin sachets and micro-XRF core-scanning
Source: Sci Rep. 2019 Apr 29;9:6601. doi: 10.1038/s41598-019-43015-x (PMC6488570; doi:10.1038/s41598-019-43015-x)
Supplement: Supplementary file 1 — Supplementary Information [file 41598_2019_43015_MOESM1_ESM.pdf]

Supplementary Information

Rapid assessment of heavy metal pollution using ion-exchange resin sachets and micro-XRF core-scanning

Jyh-Jaan Steven Huang[1,2,3,*], Sheng-Chi Lin[4], Ludvig Löwemark[1,2,**], Sofia Ya Hsuan Liou[1,2], Queenie Chang[1,5], Tsun-Kuo Chang[6], Kuo-Yen Wei[1,2], Ian W. Croudace[7]

[1]Department of Geosciences, National Taiwan University, Taipei, Taiwan.
[2]Research Center of Future Earth, National Taiwan University, Taipei, Taiwan.
[3]Institute of Geology, University of Innsbruck, Innsbruck, Austria.
[4]Center for Teaching Excellence, National Pingtung University of Science and Technology, Pingtung, Taiwan.
[5]Center of Integrative Geosciences, University of Connecticut, Connecticut, USA.
[6]Department of Bioenvironmental Systems Engineering, National Taiwan University, Taipei, Taiwan.
[7]GAU-Radioanalytical, University of Southampton, National Oceanography Centre, Southampton, United Kingdom.

*Email: huang.jyhjaan@gmail.com; Tel: +43 512 507-54396; Fax: +43 512 507-54399; Bruno Sanders Haus, Innrain 52f, room 6O-323, 6020 Innsbruck, Austria.
**Email: loewemark@gmail.com; Tel: +886(0)2-3366-2944; Fax: +886(0)2-23636095; P.O. Box 13-318, 106 Taipei, Taiwan.

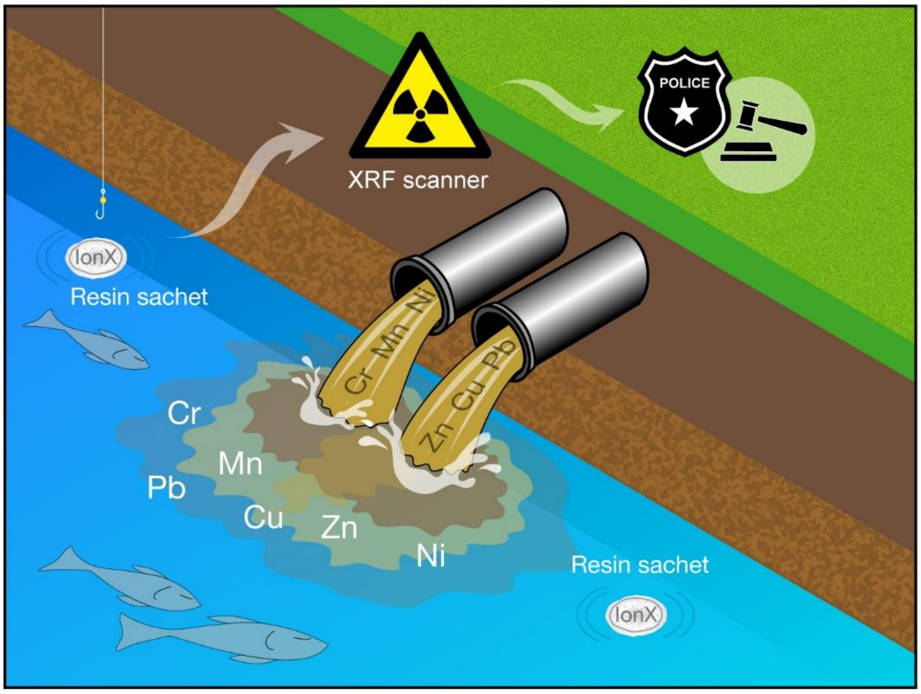

Fig. S1 Concept figure of resin sachets and XRF-CS approach.

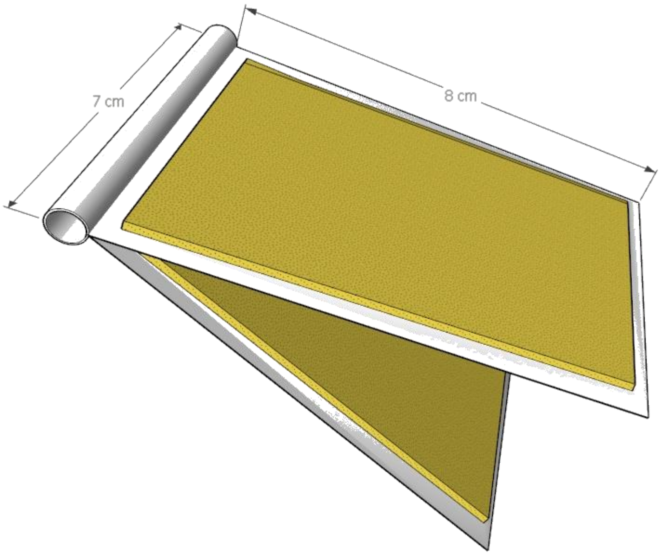

Fig. S2a Design of the resin sachet.

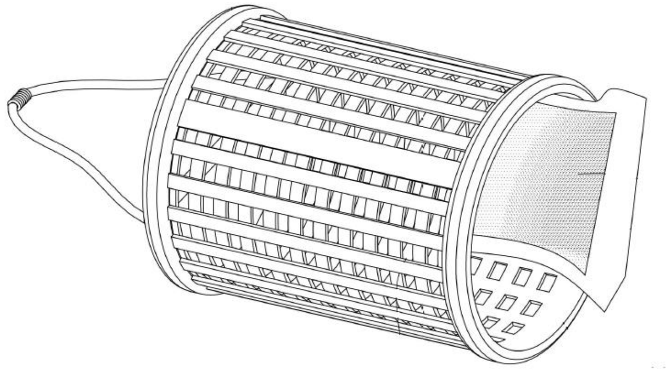

Fig. S2b Design of the field monitoring device.

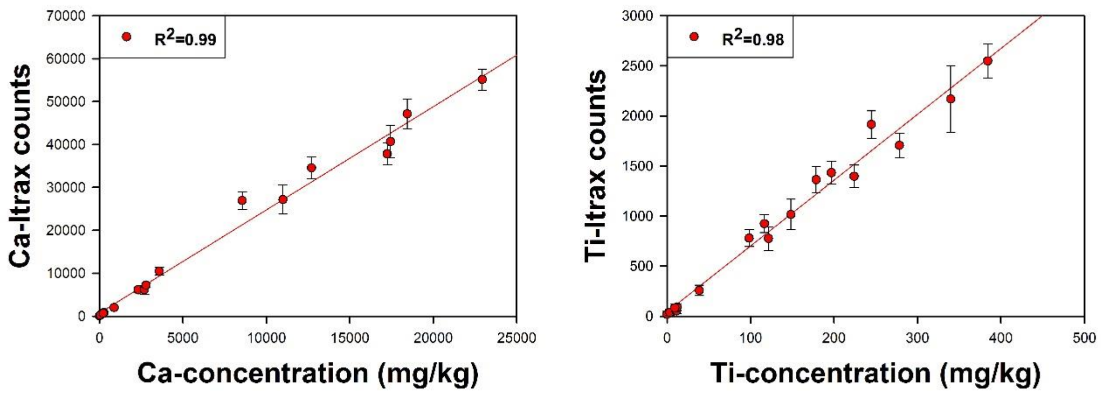

Fig. S3 Scatter plots showing linear regression lines and correlation coefficients between concentrations and XRF-CS counts (100 s exposure time, with the standard deviation of each measurement) of resin reference standards for Ca and Ti.

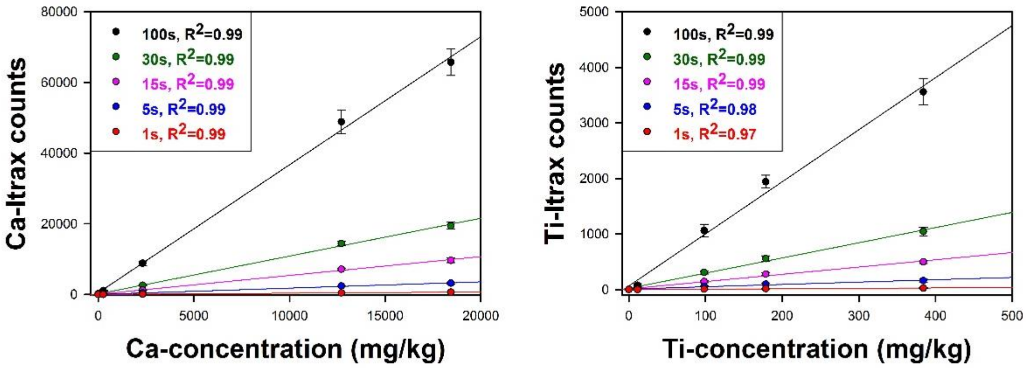

Fig. S4 Combined results of concentrations vs. XRF-CS counts for five tested exposure times. Correlation coefficients of all experiments are ≥ 0.97, suggesting exposure time has limited influence to the accuracy of XRF-CS results.

Table S1 Concentrations of standard solutions.

	Cr	Mn[#]	Ni	Cu	Zn	Pb	Ca[#]	Ti[#]
EPA effluent standard*0.1	0.2	0.2	0.1	0.3	0.5	0.1	2	0.1
EPA effluent standard*1	2	2	1	3	5	1	20	1
EPA effluent standard*10	20	20	10	30	50	10	100	5
EPA effluent standard*20	40	40	20	60	100	20	200	10

Unit: mg L^{-1}

[#]There are no specific effluent standards of Mn, Ca, Ti according to the EPA regulations of Taiwan. Ca and Ti were added to simulate natural waters.
